# Synthetic self‐adjuvanted multivalent Mucin 1 (MUC1) glycopeptide vaccines with improved in vivo antitumor efficacy

**DOI:** 10.1002/mco2.484

**Published:** 2024-02-09

**Authors:** Yang Zhou, Xinru Li, Yajing Guo, Ye Wu, Lixin Yin, Luyun Tu, Sheng Hong, Hui Cai, Feiqing Ding

**Affiliations:** ^1^ School of Pharmaceutical Sciences (Shenzhen) Shenzhen Campus of Sun Yat‐Sen University Shenzhen China

**Keywords:** cancer vaccine, MUC1 glycopeptide, multivalent strategy, self‐adjuvanted vaccine

## Abstract

The tumor‐associated glycoprotein Mucin 1 (MUC1) is aberrantly glycosylated on cancer cells and is considered a promising target for antitumor vaccines. The weak immunogenicity and low sequence homology of mouse mucins and human MUC1 are the main obstacles for the development of vaccines. Herein, a self‐adjuvanted strategy combining toll‐like receptor  2 lipopeptide ligands and T‐cell epitopes and the multivalent effect were used to amplify the immune response and evade the unpredictable immunogenicity, generating two self‐adjuvanted three‐component MUC1 vaccines (mono‐ and trivalent MUC1 vaccines). To simulate the aberrantly glycosylated MUC1 glycoprotein, the MUC1 tandem repeat peptide was bounded with Tn antigens at T9, S15, and T16, and served as B‐cell epitopes. Results showed that both vaccines elicited a robust antibody response in wild‐type mice compared with a weaker response in MUC1 transgenic mice. The trivalent vaccine did not elevate the antibody response level compared with the monovalent vaccine; however, a more delayed tumor growth and prolonged survival time was realized in wild‐type and transgenic mouse models treated with the trivalent vaccine. These results indicate that the self‐adjuvanted three‐component MUC1 vaccines, especially the trivalent vaccine, can trigger robust antitumor effects regardless of sequence homology, and, therefore, show promise for clinical translation.

## Graphical abstract

The synthetic self‐adjuvanted mono‐ and trivalent MUC1 vaccines comprising the TLR2 ligand (Pam_3_CSK_4_), T cell epitope (P30), and B cell epitope (MUC1 glycopeptide) with improved immune responses and in vivo antitumor efficacy

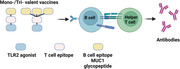

## 1 INTRODUCTION

Mucins are heavily *O*‐glycosylated proteins that are primarily produced by glandular and ductal epithelial cells and play critical roles in lubrication and protection from various exogenous and endogenous insults.^1^ Mucin 1 (MUC1), which is the first murine mucin to be identified and characterized,^2^ contains an extracellular domain with a variable number of tandem repeats (VNTRs), each composed of 20 amino acids (PDTRPAPGSTAPPAHGVTSA) with five potential sites for *O*‐glycosylation.^3^ Due to the misregulation of 1,3‐galactosyltransferase (T‐synthase) and *α‐N*‐acetylgalactosaminide *α*−2,6‐sialyltransferase‐1 (ST6GalNAc‐1),^4^ MUC1 is overexpressed in human adenocarcinomas with truncated and immature Tn/STn *O*‐glycans.^5^ These are tumor‐associated carbohydrate antigens found in clinical specimens of different types of cancers, with the Tn, T, and STn antigens being most relevant.^6^ Based on the aspects such as therapeutic function, immunogenicity, specificity, and expression level, the NCI Translational Research Working Group prioritized MUC1 as the second‐best potential target out of 75 tumor‐associated antigens for the development of cancer vaccines.^7^

As MUC1 isolated from tumor cells carries antigens typical of tumor cells as well as healthy cells, these glycoproteins are not only weakly immunogenic but can also cause severe autoimmune reactions against normal MUC1.^8^ Fully synthetic glycopeptide epitopes with different glycosylation patterns afford the development of MUC1 vaccines. The first fully synthesized vaccines mainly focused on coupling MUC1 glycopeptide with carrier proteins (such as bovine serum albumin and keyhole limpet hemocyanin), adding additional adjuvants such as Freund's adjuvant. Although high titers of specific antibodies have been induced in animals, the resulting weak overall immune response, unexpected anticarrier reactions, and intense inflammatory response caused by the added adjuvant has restricted application of these vaccines.^9^, ^10^ As a result, multicomponent approaches aimed at eliminating the carrier protein and boosting the immune response have been pursued. For example, newly developed vaccines adopted MUC1 glycopeptide as a B‐cell epitope coupled with T‐cell epitopes from different sources and/or toll‐like receptor (TLR) agonists.^8^, ^11^, ^12^, ^13^, ^14^, ^15^, ^16^ These self‐adjuvanted vaccines are state‐of‐the‐art constructs, enabling simultaneous uptake of antigens and agonists by the same antigen presenting cell and leading to enhanced antigen‐directed immune responses.^12^ Moreover, T helper (Th) cell activation evokes an essential class switch from low‐affinity and short‐lived immunoglobulin M (IgM) antibodies to high‐affinity immunoglobulin G (IgG) antibodies.^12^

Numerous studies have shown that glycosylation mode of MUC1 is crucial for immune efficacy.^17^, ^18^, ^19^ The antiserum produced by the nonglycosylated MUC1 vaccines do not specifically bind to tumor cells expressing MUC1.^20^ The MUC1 glycosylation sites and types can affect the conformation of peptide epitopes. The glycosylated MUC1 glycopeptide vaccines have high selectivity towards tumor cells, which is related to specific glycosylation patterns.^21^, ^22^ Furthermore, the glycosylation of immunodominant epitope PDTRP can greatly improve the level of immune response.^23^ The conformation of PDTRP can also be affected by the glycosylation of adjacent Ser/Thr on VNTRs and cause higher levels of immune response.^24^ There have also been reports suggesting that the STAPPA region probably induces the adoption of a helical conformation and leads to tumor selectivity^13^, ^25^; however, excessive glycosylation may hinder antigen recognition and lead to reduced immunogenicity.^13^

Based on existing research, we hypothesized that a self‐adjuvanted strategy utilizing Pam_3_‐Cys‐Ser‐Lys_4_ (Pam_3_CSK_4_) as the ligand of TLR2, the peptide P30 (TT947‐967: FNNFTVSFWLRVPKVSASHLE) derived from tetanus toxoid as a Th cell epitope, and the MUC1 tandem repeat peptide (HGVTSAPDTRPAPGSTAPPA) as a B‐cell epitope could be used to amplify the immune response and evade anticarrier immune responses. Tn antigens were designed to bind to the MUC1 tandem repeat peptide at positions T9, S15, and T16 to achieve selectivity and immune amplification. Additionally, the multivalent effect, which promoted receptor protein aggression on the cell membrane surface and led to an enhanced immune response from vaccines,^26^ was employed to obtain mono‐ and trivalent MUC1 self‐adjuvanted vaccines. As human MUC1‐transgenic (Tg) mice were considered a powerful tool for simulating human MUC1‐tolerant environments,^27^ vaccine efficacy was evaluated in both wild‐type (WT) and MUC1‐Tg mouse models. Notably, the described three‐component self‐adjuvanted vaccines were expected to exhibit effective antitumor immune responses in both mouse models, especially in the case of trivalent vaccines.

## 2 RESULTS

### 2.1 Synthesis of the three‐component mono‐ and trivalent glycopeptide immunogens

**FIGURE 1:**
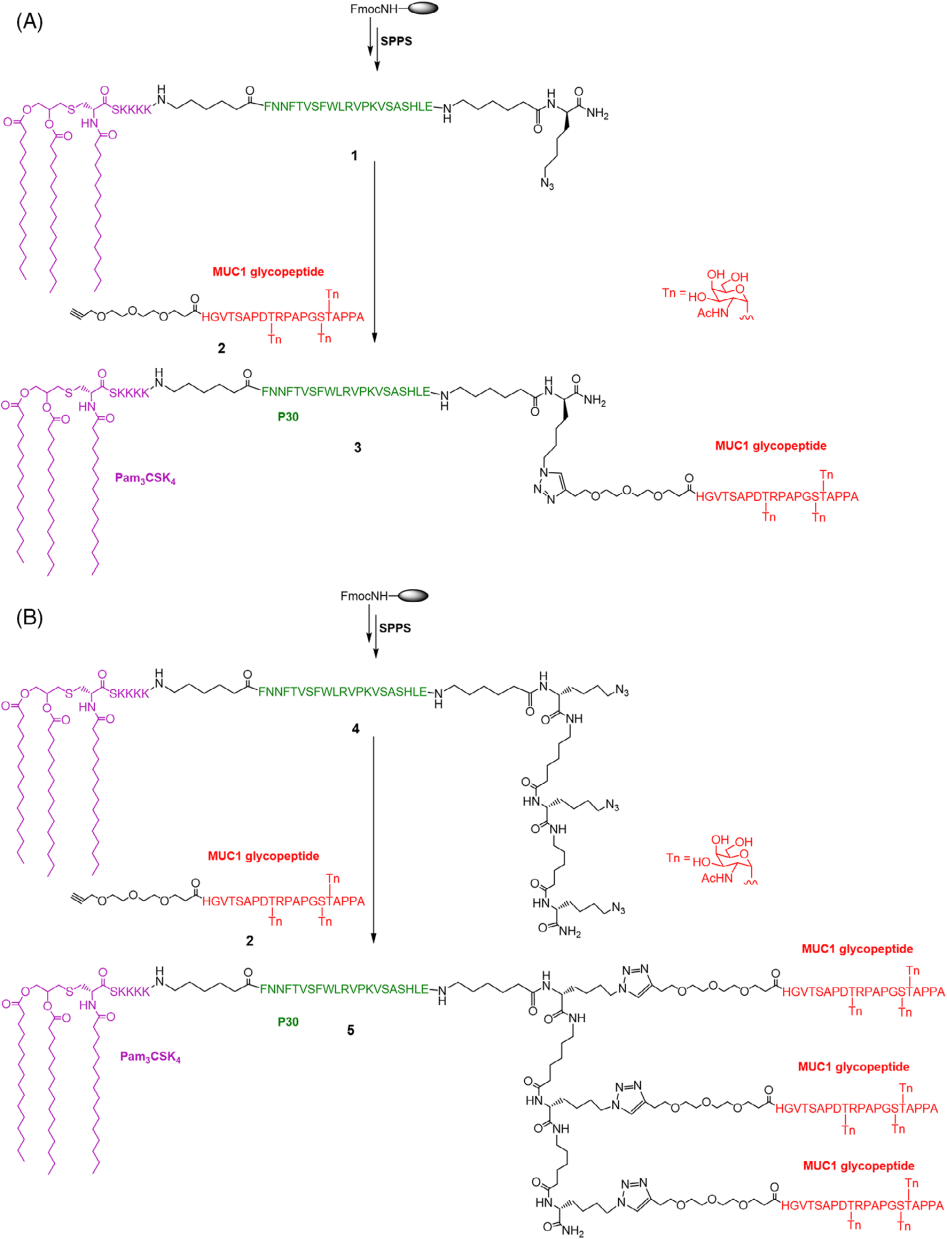
Schematic illustration of synthesis of self‐adjuvanted multivalent Mucin 1 (MUC1) glycopeptides. Synthesis of three‐component monovalent (A) and trivalent (B) glycopeptide immunogens.

We previously applied copper (I)‐catalyzed alkyne‐azide 3 + 2 cycloaddition (using click chemistry) to synthesize a trivalent, three‐component HIV‐1 V3 glycopeptide immunogen, which consisted of a 33‐mer V3 glycopeptide epitope, a universal Th epitope P30, and a lipopeptide (Pam_3_CSK_4_) functioned as a ligand of TLR2.^28^ The synthesis of the mono‐ and trivalent three‐component MUC1 immunogen followed a similar strategy (Figure 1).

**FIGURE 2:**
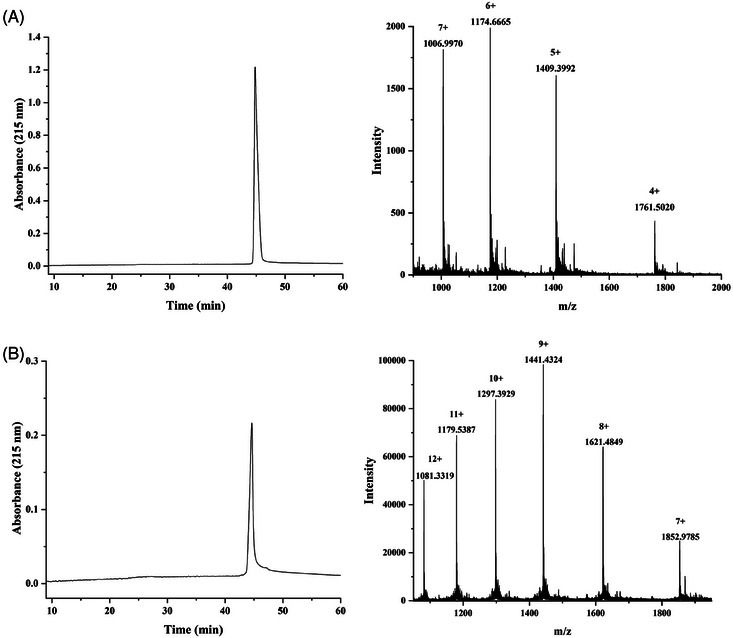
Characterization of self‐adjuvanted multivalent MUC1 glycopeptides. High‐performance liquid chromatography (HPLC) and high‐resolution mass spectrometry (HRMS) analysis of the synthetic three‐component mono‐ and trivalent glycopeptide immunogens. (A) Three‐component monovalent glycopeptide 3. (B) Three‐component trivalent glycopeptide 5. Left panel, the analytical HPLC profile; right panel, the HRMS spectra. Analytical HPLC was run on a CN column using a linear gradient of 20−80% acetonitrile containing 0.1% trifluoroacetic acid (TFA) over 60 min. The HRMS analysis was performed on an Orbitrap Fusion Lumos Tribrid Mass Spectrometer.

First, one or three Fmoc‐Lys(N_3_)‐OH moieties were installed at the C‐terminus during solid phase peptide synthesis, followed by P30 T‐cell epitope FNNFTVSFWLRVPKVSASHLE installation and Pam_3_CSK_4_ lipopeptide attachment. To minimize the potential steric congestion around the different components and favor antigen accessibility and presentation,^12^ the 6‐aminohexanoic acid residues were placed to function as flexible spacers as was commonly used for vaccine development.^29^, ^30^, ^31^ The crude peptide was cleaved from the resin using cocktail R solution (trifluoroacetic acid/triisopropylsilane/water = 90:5:5) and then purified on a polar‐CN column for mono‐ and trivalent lipopeptide scaffolding, as previously reported.^28^ In parallel, the glycopeptide antigen **2** with three Tn antigens linked at T9, S15, and T16 of the MUC1 domain HGVTSAPDTRPAPGSTAPPA was synthesized in solid phase and N‐terminally acylated with an alkyne‐functionalized spacer, carboxylic acid, as described previously.^23^ The Tn antigens were synthesized according to our previous method and incorporated as building blocks onto the peptide skeleton.^32^ Subsequently, the glycopeptide was released from the resin, and the protecting groups were removed from the carbohydrate portions. After purification by high‐performance liquid chromatography (HPLC), the glycopeptide was conjugated to the mono‐ and triazido‐functionalized lipopeptides (**1** and **4**) via click chemistry to obtain the mono‐ and trivalent glycopeptide‐lipopeptide vaccine candidates. The purity and structure of these glycopeptides were confirmed by analytical HPLC and high‐resolution mass spectrometry (HRMS), respectively (Figure 2).

### 2.2 Immunological evaluation of mono‐ and trivalent MUC1 vaccines

**FIGURE 3:**
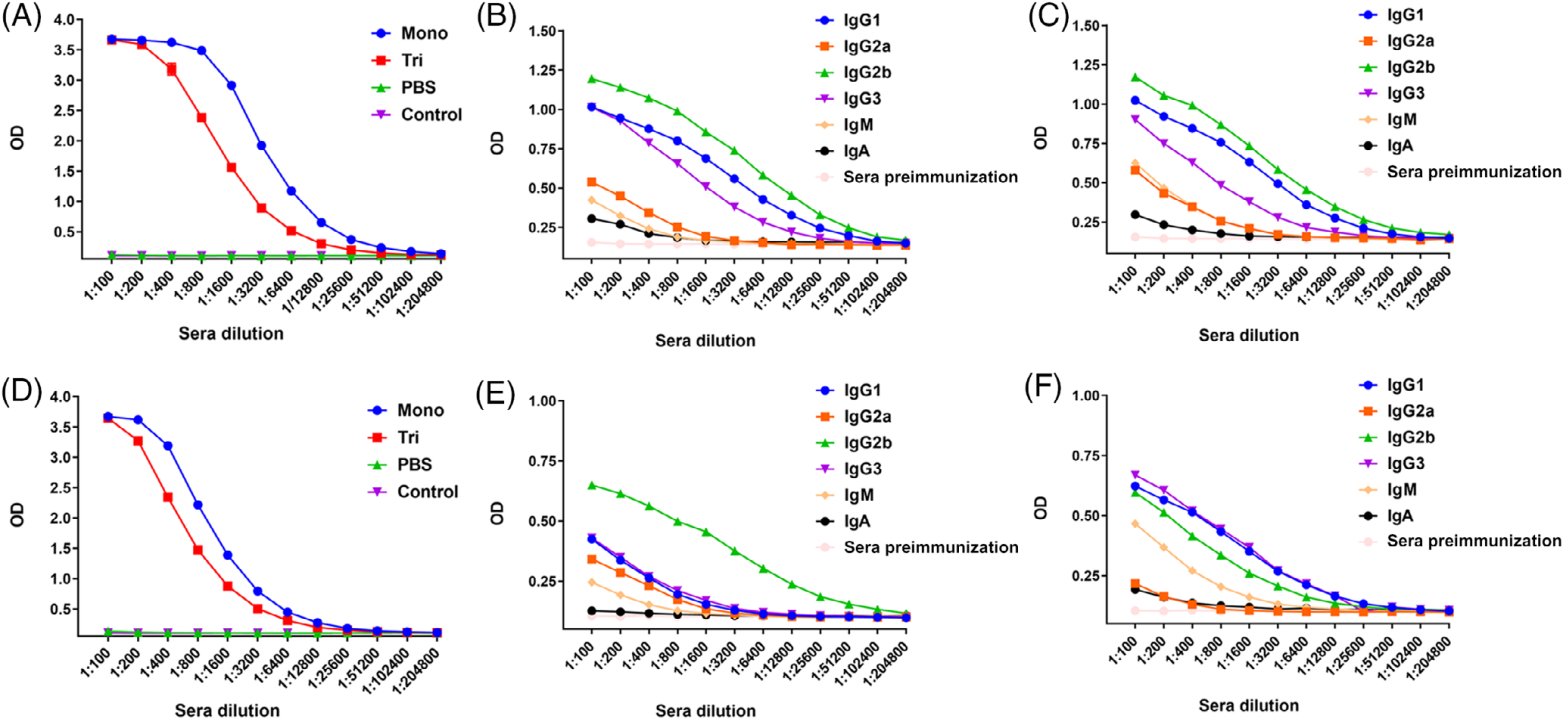
Self‐adjuvanted multivalent MUC1 glycopeptide vaccines strongly elicited the production of antibodies. Total IgG antibody (A) and antibody isotype analysis of the antisera induced by the monovalent glycopeptide (B) and trivalent glycopeptide (C) in wild‐type (WT) mice. Total IgG antibody (D) and antibody isotype analysis of the antisera induced by the monovalent glycopeptide (E) and trivalent glycopeptide (F) in MUC1 transgenic (Tg) mice.

Following the successful synthesis of the mono‐ and trivalent MUC1 vaccines, we evaluated their immune responses induced in mice. Female C57BL/6 mice (*n* = 5 per group) were subcutaneously immunized five times at intervals of 2 weeks (day 1, 14, 28, 42, and 56). The dosage was calculated based on the amount of MUC1 peptide, and both mono‐ and trivalent vaccines were administered with 8 µg of MUC1 glycopeptide per mouse. A control group was immunized with phosphate‐buffered saline (PBS; 100 µL per mouse). One week after the fifth immunization, sera were collected to evaluate the antibody responses using enzyme‐linked immunosorbent assays (ELISA). We detected the total IgG against MUC1 glycopeptide. As shown in Figure 3A, both the mono‐ and trivalent vaccines strongly induced the production of antibodies specifically against the MUC1 glycopeptide, whereas the monovalent vaccine appeared to induce higher antibody concentrations. We then used goat‐anti mouse IgG1, IgG2a, IgG2b, IgG3, IgM, and Immunoglobulin A (IgA) as the secondary antibodies and alkaline phosphatase‐conjugated donkey‐anti goat IgG as the third antibody to analyze the antibody isotypes. As shown in Figures 3B and C, the administration of the mono‐ and trivalent vaccines mainly induced the production of IgG2b, IgG1, and IgG3, and the apparent increase in IgG2a, IgM, and IgA indicated an effectively specific immune response against the MUC1 glycopeptide. The high production of IgG2b showed that a T‐cell‐independent response was induced.^33^ The significantly enhanced expression of IgG1 indicated strong antibody‐dependent cell‐mediated cytotoxicity and/or antibody‐dependent cellular phagocytosis due to its strong affinity with activating Fcγ receptors (FcγRs).^34^ IgG3 is considered a carbohydrate antigen‐associated isotype.^35^ The increase in IgG1 and IgM concentrations further suggest that effective immune memory was established.^36^

Encouraged by the promising results and to simulate the MUC1 response in humans, we further tested the vaccines in MUC1‐Tg mice. Total IgG showed a significant immune response induced by both the mono‐ and trivalent vaccines, as shown in Figure 3D. Similarly, the highest concentration of IgG2b was induced by the monovalent vaccine, and a similar expression of IgG1 and IgG3 was observed for the Th2 and Th1 isotypes, respectively, suggesting a mixed Th1/Th2 response was induced by the trivalent candidate (Figures 3E and F).^37^

**FIGURE 4:**
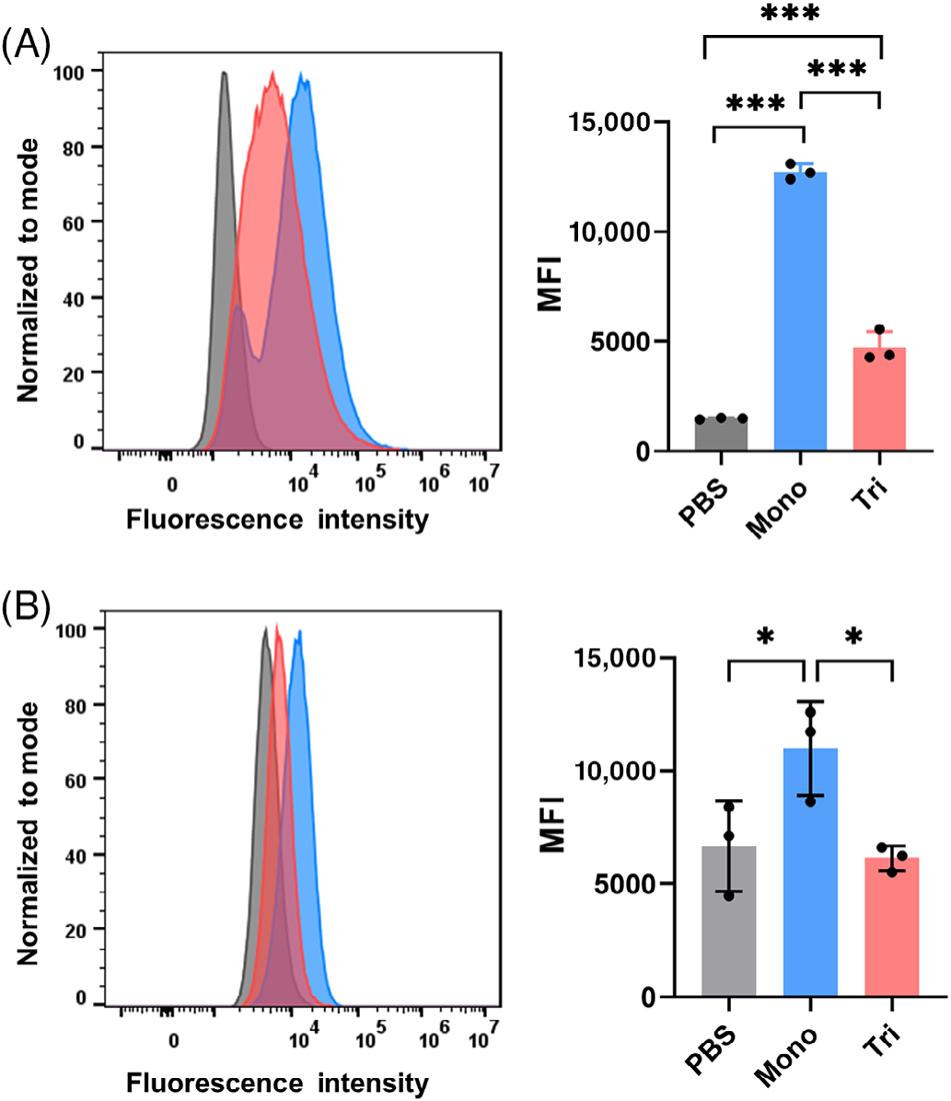
Antibodies from the vaccinated mice specifically bound to MUC1‐expressed B16‐MUC1 cells. Flow cytometry analysis of the binding ability of the antisera (1:100 dilution) from WT (A) and Tg (B) mice to B16‐MUC1 cells. PBS: incubation of the cells with sera from the mice that were not immunized.

We then investigated the binding ability of anti‐MUC1 antibodies from the vaccinated mice to MUC1‐expressed B16‐MUC1 cells using fluorescence‐activated cell sorting (FACS). B16‐MUC1 cells were incubated with both WT (Figure 4A) and Tg (Figure 4B) mice antisera (1:100 dilution) and then with goat anti‐mouse IgG antibody conjugated to AlexaFluor488 as a secondary antibody. The sera antibody‐recognized cells labeled by fluorescence were then captured and counted via FACS analysis. As shown in Figure 4, the sera induced by the monovalent vaccine showed a stronger binding ability than that elicited by the trivalent candidate, which was consistent with total IgG results. In contrast, the antisera showed no specific binding ability to HEK293T cells (Figure S1), which was used as a negative control.

### 2.3 Evaluation of antitumor immune activity in a solid tumor model of WT mice

**FIGURE 5:**
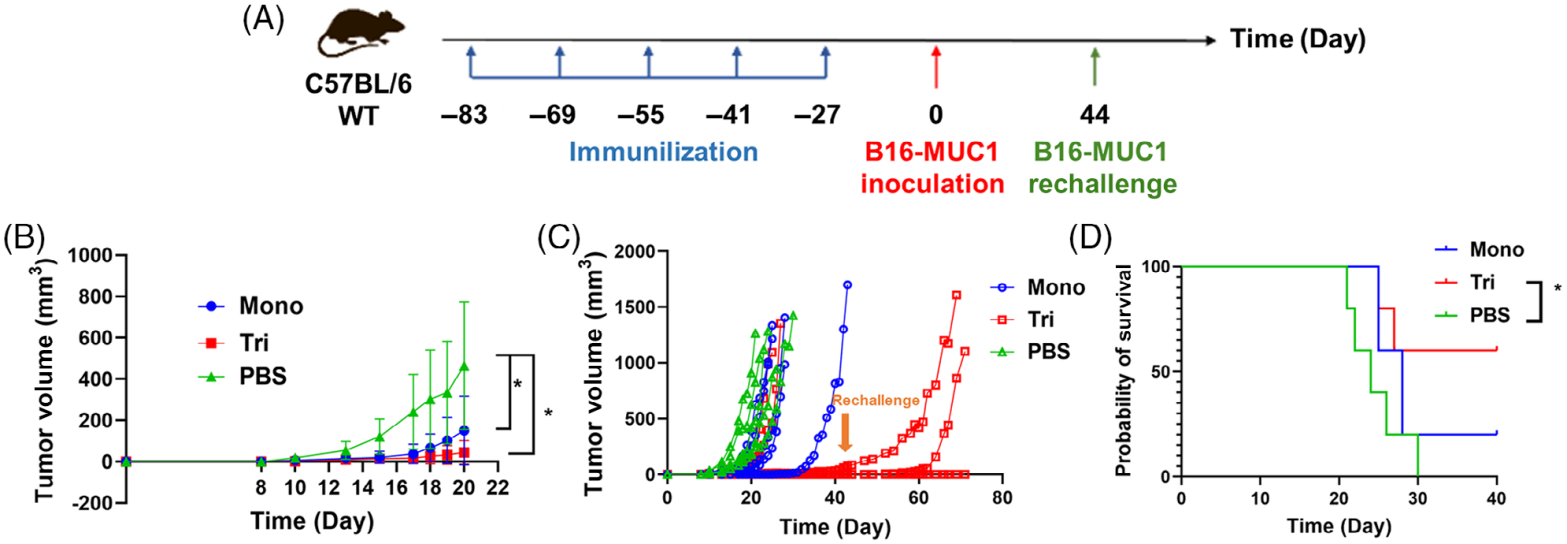
Self‐adjuvanted multivalent MUC1 glycopeptide vaccines induced effective antitumor immune responses in a solid tumor model of WT mice. Schedule of immunization and tumor inoculation of WT C57BL/6 mice (A). Tumor growth curves (B). Mean tumor volumes and standard errors of the mean are shown (*n* = 5). Statistical analysis using one‐way analysis of variance (ANOVA): *, *p* < 0.05. Tumor growth kinetics (C). Survival curves (D). Statistical analysis using a Log‐Rank (Mantel–Cox) test: *, *p* < 0.05.

To further characterize the potential therapeutic activity of the two candidate vaccines, we explored the antitumor activity in vivo in the B16‐MUC1 murine model. Twenty‐seven days after the fifth immunization, female C57BL/6 mice were subcutaneously inoculated with B16‐MUC1 cells in the right flank (2.5 × 10^5^ cells/mouse, *n* = 5), and the tumor volume was monitored every other day (Figure 5A). Repeat injection of the two vaccines showed no significant biocompatibility threats compared with the nonimmunized group. The results suggested that the mono‐ and trivalent vaccines delayed the mean tumor‐growth process (Figure 5B) and prolonged the survival time of mice (Figure 5D). Three mice from the trivalent group lived more than 40 days with apparently delayed tumor growth, and one mouse had no tumor growth at all (Figure 5C). Accordingly, we rechallenged B16‐MUC1 cells in these three mice on the 71st day after final immunization. The tumor rechallenges still failed to induce tumor growth in these mice. These results indicated that the two vaccines could potentially induce strong and effective immune memory to defend against the attack of tumor cells in vivo for a long period.

### 2.4 Evaluation of antitumor immune activity in a solid tumor model of MUC1‐Tg mice

**FIGURE 6:**
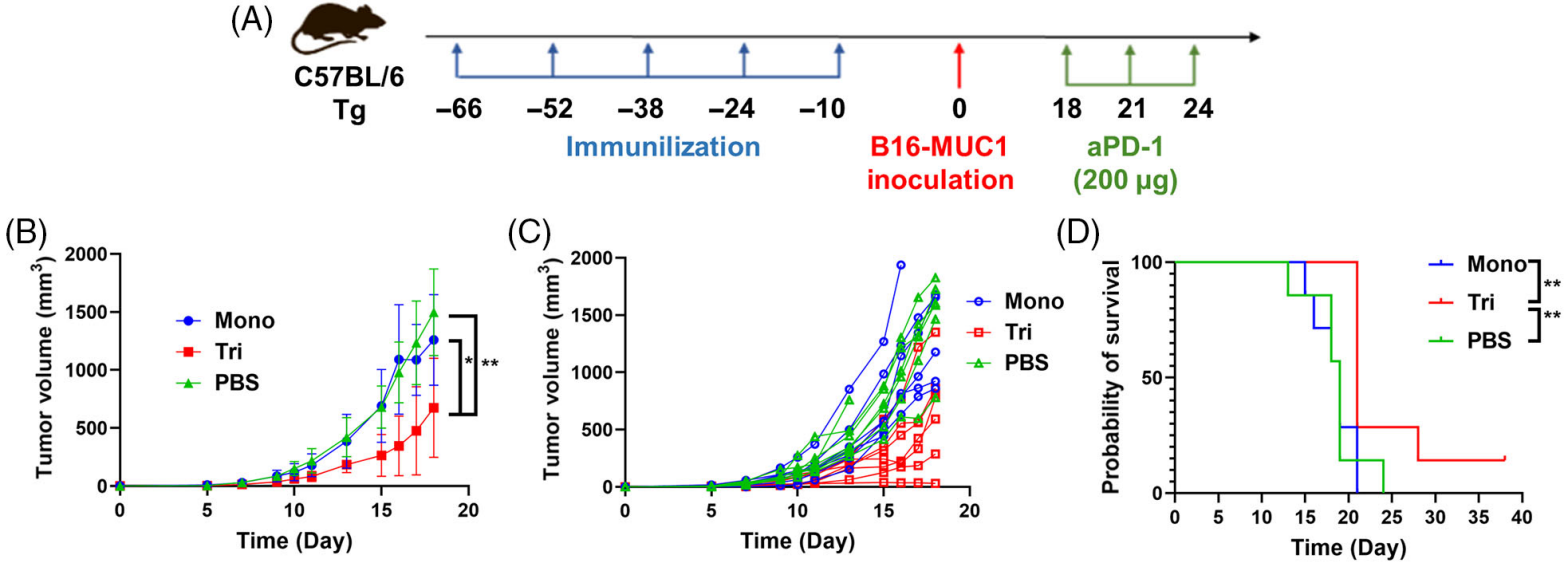
Self‐adjuvanted multivalent MUC1 glycopeptide vaccines induced effective antitumor immune responses in a solid tumor model of MUC1‐Tg mice. Schedule of immunization and tumor inoculation of MUC1 Tg C57BL/6 mice (A). Tumor growth curves (B). Mean tumor volumes and standard errors of the mean are shown (*n* = 5). Statistical analysis via one‐way analysis of variance (ANOVA): *, *p* < 0.05; **, *p* < 0.01. Tumor growth kinetics (C). Survival curves (D). Statistical analysis using a Log‐Rank (Mantel–Cox) test: **, *p* < 0.01.

Based on the promising results in WT mice and the low sequence homology between mice and human mucins, we subsequently performed the same experiments using human MUC1‐Tg mice. Ten days after the fifth immunization, we implanted 2.5 × 10^5^ B16‐MUC1 cells subcutaneously to construct the solid tumor model of MUC1‐Tg mice (Figure 6A). Programmed cell death 1 ligand 1 (PD‐L1) is highly expressed on the surface of many tumors and is a cosuppressor of immune responses.^38^ Studies have shown that tumor cells expressing MUC1 exhibit upregulation of PD‐L1, and downregulation of MUC1 and PD‐L1 can exert a synergistic effect, demonstrating superior antitumor effects.^39^, ^40^, ^41^ Therefore, PD‐1 antibody (aPD‐1) for blocking the interaction between PD‐1 and PD‐L1 was administered intraperitoneally to the mice on day 18, 21, and 24 (200 µg per mouse). Although the tumor growth was faster in the Tg mice compared with the WT mice, the trivalent vaccine significantly delayed tumor growth compared with the control group and monovalent candidate, and also improved the mouse survival rate when combined with aPD‐1 (Figures 6B, C, and D).

## 3 DISCUSSION

Successful vaccines tend to be multifunctional, and self‐adjuvanted multicomponent vaccines can achieve many functions within a limited structure. The self‐adjuvanted MUC1 vaccines that have been previously reported mainly include two‐ and three‐component vaccines incorporating MUC1 glycopeptide, T‐cell epitopes, and/or adjuvants such as TLR agonist.^7^, ^12^, ^13^, ^14^, ^15^, ^23^, ^24^ T‐cell epitopes include the peptides P2, P4, and P30 derived from the tetanus toxoid,^42^, ^43^ and the Th cell peptide from ovalbumin (OVA_323–339_).^10^ Pam_3_CSK_4_ is the most commonly used adjuvant.^7^, ^16^ The differences between MUC1 glycopeptides are reflected in glycosylation sites (T4, S5, T9, S15, and T16)^13^ and types (Tn, T and STn).^16^

Here, we synthesized three‐component multivalent self‐adjuvanted vaccines comprising Pam_3_CSK_4_, P30, and MUC1 glycosylated at T9, S15 and T16 with Tn, and studied their immune effects in Tg mice. Although the structures proposed by Boons and cowokers,^7^ Corzana and cowokers,^24^ and Li and workers^43^ share some similarities with the vaccines, the MUC1 glycopeptide sequence, glycosylation pattern, and the types of TLR agonists are varied, resulting in different levels of immune responses. In one of our previous works,^44^ a similar structure induced high levels of specific antibodies, but the effect of the vaccine in Tg mice was not studied. In addition, we introduced a multivalent strategy to improve the immune response as the multivalent vaccines were reported to be more effective than the monovalent vaccine.^23^ Structures of both vaccines are slightly different from our previously reported mono‐, di‐, and tetravalent MUC1 vaccines, mainly reflected in the glycosylation patterns and T‐cell epitopes.^23^ However, both caused a similarly lower antibody level in the multivalent group compared with that of the mono‐ and divalent groups. A better antitumor effect was inconsistently induced by the trivalent vaccine. This can be partly explained in terms of antibody subtypes. Specifically, in Tg mice, IgG1 levels in the trivalent group were much higher than IgG2a, leading to a higher Th2 immune response.^45^ And the IgG2a levels in the trivalent group were lower than the monovalent one. With the same dose of MUC1 administration, the trivalent group received relatively less T‐cell epitopes and adjuvants, structurally explaining the Th1/Th2 immune response. The higher levels of IgG3 and IgM in the trivalent group relative to the monovalent group may also indicate that its immune response is primarily caused by MUC1 glycopeptides.^17^ This may result in the elicited immune response acting more specifically on tumor cells.^46^ This difference in antibody subtypes was not observed in the WT mice, possibly because their immune systems also recognized tumor cells as foreign, whereas the immune systems of the Tg mice mainly targeted the MUC1 glycopeptide vaccine.^1^ This further illustrates the superiority and necessity of Tg mouse models for the development of clinical drugs. The more biased Th2 immune response in the trivalent group may lead to more cytokine secretion and, therefore, elicit a more potent antitumor effect.^47^ In addition, given the unique structural characteristics of the two self‐adjuvanted multicomponent vaccines, we propose that the vaccines self‐assemble in vitro or undergo self‐assembly in vivo upon binding to the macrophage galactose‐type lectin (MGL) receptors,^14^ and that the different self‐assembly properties of them lead to differences in antitumor effects. As MUC1 is overexpressed on the surface of tumors, the multivalent vaccine may better mimic the form in which MUC1 is present in the tumors, thereby inducing specific high levels of immune responses and better antitumor effects.

Although there are still some unresolved issues like the conformation of the two vaccines, the fact that the antitumor effects of them have been enhanced in a number of ways and validated in both WT and more human‐adapted Tg mice models suggests that they are vaccines with clinical potential and provides an idea for the development of an effective antitumor vaccine.

## 4 MATERIALS AND METHODS

### 4.1 Cells and mice

B16‐MUC1 and HEK293T cells were obtained from BMCR and maintained in Dulbecco's modified eagle medium (Gibco) supplemented with 10% fetal bovine serum and 1% penicillin/streptomycin. The cells were incubated at 37°C in a 5% carbon dioxide atmosphere.

Female C57BL/6 mice were obtained from GemPharmatech Co., Ltd. and were bred in‐house at the School of Pharmaceutical Sciences (Shenzhen), Sun Yat‐sen University, Guangzhou, Guangdong, China. The animals were housed in groups with unlimited access to food and water. All mouse studies were performed in compliance with the Institutional Animal Care and Use Committee of Sun Yat‐sen University.

### 4.2 Peptide synthesis

Peptides were synthesized by standard solid phase synthesis^11^, ^43^ of Fmoc chemistry with Nα Fmoc‐amino acids (GL Biochem Ltd., Shanghai). The peptides were synthesized at the 0.1 mmol scale and prolonged starting from H‐Ala‐2‐Cl‐Trityl resin with a loading of 0.13 mmol/g. Fmoc removal was executed using a solution of 20% piperidine in N,N‐dimethylformamide (DMF). Coupling reactions of Fmoc amino acids and spacer were carried out by activation with 2‐(1H‐benzotriazole‐1‐yl)−1,1,3,3‐tetramethylhexafluorophosphate (HBTU)/N‐hydroxybenzotriazole (HOBt) using N,N‐diisopropylethylamine (DIEA) in N‐Methylpyrrolidone (NMP). The Fmoc amino acids (6.0 equiv.), HBTU (6.0 equiv.), HOBt (6.0 equiv.), and DIPEA (12 equiv.) were added automatically. The protected Tn glycosyl Ser/Thr building blocks were activated by O‐(7‐azabenzotriazole‐1‐yl)‐N,N,N’,N’‐tetramethyluronium hexafluorophosphate (HATU)/N‐hydroxy‐7‐azabenzotriazole (HOAt) using DIEA in NMP, while the glycosyl amino acid building blocks (2.0 equiv.), HATU (2.5 equiv.), HOAt (2.5 equiv.), and DIEA (5.0 equiv.) were dissolved in NMP and mixed manually with the resin. After coupling of all the building blocks, the resin was transferred from the peptide synthesizer into a flask. The resin was treated with a mixture of TFA/TIS/H_2_O (90/5/5, v/v/v) for 2 h to detach the peptides. The crude glycopeptides were purified by HPLC. After lyophilization, the glycopeptides with Tn were deprotected by treatment with a solution of 1% MeONa in MeOH (pH 10.0). Then, the target glycopeptides were obtained after purification and lyophilization.

### 4.3 Peptide purification

Analytical reverse‐phase HPLC was performed on a Waters e2695 HPLC system equipped with a dual absorbance UV detector. All the MUC1 peptides and glycopeptides were run on a C18 column (YMC‐Triart C18; 4.6 × 250 mm, 5 µm) at a flow rate of 1 mL/min using a linear gradient of 10−30% acetonitrile containing 0.1% trifluoroacetic acid for 20 min. The lipopeptides were run on a CN column (YMC‐Pack CN; 4.6 × 250 mm, 5 µm) using a linear gradient of 20−80% acetonitrile containing 0.1% trifluoroacetic acid for 60 min. HRMS spectra were measured on an Orbitrap Fusion Lumos Tribrid Mass Spectrometer. For the MUC1 peptides and glycopeptides, preparative reverse‐phase HPLC was performed on a Waters 2489 HPLC system equipped with a dual absorbance UV detector using a C18 column (Waters SymmetryPrep™; 19 × 300 mm, 7 µm) at a flow rate of 20 mL/min. The preparative HPLC purification of the lipopeptide was performed on a CN column (YMC‐Pack CN; 10 × 250 mm, 5 µm).

### 4.4 Synthesis of three‐component monovalent immunogen 3

Lipopeptide **1** (0.5 mg, 0.11 µmol), MUC1 alkyne glycopeptide **2** (0.4 mg, 0.15 µmol), tris‐hydroxypropyltriazolylmethylamine (THPTA; 0.2 mg, 0.46 µmol), and catalytic equivalent CuOAc were dissolved in 50 µL of water. The mixture was stirred at 40°C for 18 h. The reaction mixture was diluted with 1 mL water and then lyophilized. The crude product was purified using RP‐HPLC on a CN column to obtain immunogen **3** (0.39 mg, 50%). HRMS: calculated, *M* = 7042.9724; found (*m*/*z*): 1006.9970 [M+7H]^7+^, 1174.6665 [M+6H]^6+^, 1409.3992 [M+5H]^5+^, and 1761.5020 [M+4H]^4+^. RP‐HPLC retention time, *t*_R_ = 44.8 min.

### 4.5 Synthesis of three‐component trivalent immunogen 5

Lipopeptide **4** (0.5 mg, 0.1 µmol), MUC1 alkyne glycopeptide **2** (1.1 mg, 0.4 µmol), THPTA (0.4 mg, 0.92 µmol), and catalytic equivalent CuOAc were dissolved in 50 µL water. The mixture was incubated at 40°C for 18 h. The reaction mixture was diluted with 1 mL water and then lyophilized. The crude product was purified via RP‐HPLC on a CN column to obtain immunogen **5** (0.74 mg, 57%). HRMS: calculated, *M* = 12963.8388; found (*m*/*z*): 1081.3319 [M+12H]^12+^, 1179.5387 [M+11H]^11+^, 1297.3929 [M+10H]^10+^, 1441.4324 [M+9H]^9+^, 1621.4849 [M+8H]^8+^, and 1852.9785 [M+7H]^7+^; *t*_R_ = 44.3 min.

### 4.6 Immunization

Female SPF C57BL/6 mice aged 6−8 weeks were randomly divided into three groups with five mice in each group. The mice were immunized subcutaneously every 2 weeks with mono‐ (24 µg per immunization) or trivalent vaccines (14 µg per immunization) diluted in PBS (pH = 7.4) for five times. The sera were collected via retro‐orbital bleeding after the final vaccination and then centrifuged at 587 g for 30 min for serum separation. The MUC1‐Tg mice were subjected to the same protocol with seven mice in each group.

### 4.7 ELISA to determine antibody titers and isotypes

Sera collected on day 70 were pooled by group and analyzed using ELISA. The 96‐well ELISA microtiter plates were coated with 5 µg/mL of Neutravidin in PBS (100 µL/well) and incubated at 4°C overnight. The plates were washed with washing buffer (PBS/0.05% Tween‐20) and blocked with 2% bovine serum albumin (w/v) in PBS at room temperature for 1 h. After washing three times, 2 µg/mL of the biotinylated glycopeptide (glycosylated MUC1 peptide coupling with *D*‐biotin via solid phase peptide synthesis; Figure S2) dissolved in 1% bovine serum albumin (100 µL/well) was added and incubated at 37°C for 1 h. After washing the plates with washing buffer, the sera were diluted with coating buffer in two‐fold serial dilutions starting from 1:100 in triplicate (100 µL/well). After incubating for 2 h at room temperature, the plates were washed with washing buffer four times and then incubated with alkaline phosphatase‐labeled goat anti‐mouse IgG (Invitrogen, Thermo Fisher Scientific) diluted at a ratio of 1:3000 in PBS (100 µL/well) at room temperature for 1 h. After washing, 1‐step para‐nitrophenyl phosphate substrate (Thermo Fisher Scientific) was added (100 µL/well) and the reaction was stopped after 15 min by adding 2 M sodium hydroxide (50 µL/well). The absorbance at 405 nm was then determined using a Tecan microplate reader.

The distribution of IgG1, IgG2a, IgG2b, IgG3, IgA, and IgM in the sera was determined via ELISA using a similar procedure as described for the peptide‐specific antibody detection. After the sera were added to the plates and incubated, goat anti‐mouse IgG1, IgG2a, IgG2b, IgG3, IgM, and IgA isotype antibodies were added (Sigma–Aldrich). After washing the plates, alkaline phosphatase‐labeled donkey anti‐goat IgG (Sigma–Aldrich) was added. All other steps were as described for the peptide ELISA.

### 4.8 Flow cytometry

B16‐MUC1 cells were cultured as previously described and harvested. The cells were then washed with FACS buffer (PBS containing 1% fetal bovine serum) and resuspended in 200 µL of FACS buffer in a 1.5‐mL Eppendorf tube at 500,000 cells/tube in triplicate. The cells were then incubated for 1 h at 4°C with mouse serum diluted by 1:100 in FACS buffer. After washing twice with FACS buffer, the cells were stained with a goat anti‐mouse IgG antibody conjugated to AlexaFluor 488 (Thermo Fisher Scientific) diluted at 1:1000 in FACS buffer. The cells were again washed twice with FACS buffer and resuspended in 500 µL FACS buffer for immediate analysis using a BD Accuri C6 plus flow cytometer and FlowJo v10.8.1 software.

### 4.9 In vivo tumor challenging

The mouse melanoma cell line B16‐MUC1 was cultured and harvested as described above. After washing with PBS, the cells were resuspended in the medium at a concentration of 2.5 × 10^6^/mL and 100 µL was subcutaneously injected into the right flank of each mouse (*n* = 5) for 14 d after the fifth immunization. Tumor volumes were monitored and recorded every other day. The tumor size was measured using digital calipers and calculated as follows: (width^2^ × length)/2. When the tumor size reached 1000 mm^3^, the mice were euthanized. Similar strategies were used on the Tg mice.

### 4.10 Statistical analysis

Data reported in the figures were analyzed and charts were generated using Prism 5 (GraphPad Software). Statistical significance was determined using two‐ or one‐way analysis of variance. Survival data were compared using Mantel–Cox (log‐rank) and/or Gehan–Breslow–Wilcoxon tests. In the figures, asterisks represent the following *p* values: *p < 0.05, **p < 0.01, and ***p < 0.001.

## AUTHOR CONTRIBUTIONS

*Vaccine synthesis and manuscript writing*: Y. Z. *Biological experiments and manuscript writing*: X. L. *Assisted in biological experiments*: Y. G., Y. W., L. Y., and L. T. *Technical support*: F. D. and S. H. *Study design, project supervision, and manuscript revision*: H. C. All the authors have provided their consent to publish this study. All authors have read and approved the final manuscript.

## CONFLICT OF INTEREST STATEMENT

The authors declare they have no conflicts of interest.

## ETHICS STATEMENT

Human subjects were not used in this study. All preclinical investigations involving animals were carried out in line with ethical standards and according to the approved protocol by Experimental Animal Ethics Committee of Sun Yat‐sen University (approval number SYSU‐YXYSZ20220317).

## Supporting information

Supporting Information

## Data Availability

The dataset generated during the current study is available from the corresponding author on reasonable request.

## References

[1] Joshi S , Kumar S , Bafna S , et al. Genetically engineered mucin mouse models for inflammation and cancer. Cancer Metastasis Rev. 2015;34(4):593‐609. 25634251 10.1007/s10555-015-9549-1 PMC4520780

[2] Spicer AP , Parry G , Patton S , et al. Molecular cloning and analysis of the mouse homologue of the tumor‐associated mucin, MUC1, reveals conservation of potential O‐glycosylation sites, transmembrane, and cytoplasmic domains and a loss of minisatellite‐like polymorphism. J Biol Chem. 1991;266(23):15099‐15109. 1714452

[3] Fang T , Van Elssen C , Duarte JN , et al. Targeted antigen delivery by an anti‐class II MHC VHH elicits focused alphaMUC1(Tn) immunity. Chem Sci. 2017;8(8):5591‐5597. 28970938 10.1039/c7sc00446j PMC5618788

[4] Ju T , Lanneau GS , Gautam T , et al. Human tumor antigens Tn and sialyl Tn arise from mutations in Cosmc. Cancer Res. 2008;68(6):1636‐1646. 18339842 10.1158/0008-5472.CAN-07-2345

[5] Radhakrishnan P , Dabelsteen S , Madsen FB , et al. Immature truncated O‐glycophenotype of cancer directly induces oncogenic features. Proc Natl Acad Sci USA. 2014;111(39):E4066‐E4075. 25118277 10.1073/pnas.1406619111 PMC4191756

[6] Sanz‐Martinez I , Pereira S , Merino P , et al. Molecular recognition of GalNAc in mucin‐type O‐glycosylation. Acc Chem Res. 2023;56(5):548‐560. 36815693 10.1021/acs.accounts.2c00723 PMC9996832

[7] Lakshminarayanan V , Thompson P , Wolfert MA , et al. Immune recognition of tumor‐associated mucin MUC1 is achieved by a fully synthetic aberrantly glycosylated MUC1 tripartite vaccine. Proc Natl Acad Sci USA. 2012;109(1):261‐266. 22171012 10.1073/pnas.1115166109 PMC3252914

[8] Gaidzik N , Westerlind U , Kunz H . The development of synthetic antitumour vaccines from mucinglycopeptide antigens. Chem Soc Rev. 2013;42(10):4421‐4442. 23440054 10.1039/c3cs35470a

[9] Keil S , Claus C , Dippold W , et al. Towards the development of antitumor vaccines: a synthetic conjugate of a tumor‐associated MUC1 glycopeptide antigen and a tetanus toxin epitope. Angew Chem Int Ed. 2001;40(2):366‐369. 10.1002/1521-3773(20010119)40:2<366::AID-ANIE366>3.0.CO;2-J 29712404

[10] Dziadek S , Kowalczyk D , Kunz H . Synthetic vaccines consisting of tumor‐associated MUC1 glycopeptide antigens and bovine serum albumin. Angew Chem Int Ed Engl. 2005;44(46):7624‐7630. 16247814 10.1002/anie.200501593

[11] Huang ZH , Shi L , Ma JW , et al. A totally synthetic, self‐assembling, adjuvant‐free MUC1 glycopeptide vaccine for cancer therapy. J Am Chem Soc. 2012;134(21):8730‐8733. 22587010 10.1021/ja211725s

[12] Pifferi C , Aguinagalde L , Ruiz‐de‐Angulo A , et al. Development of synthetic, self‐adjuvanting, and self‐assembling anticancer vaccines based on a minimal saponin adjuvant and the tumor‐associated MUC1 antigen. Chem Sci. 2023;14(13):3501‐3513. 37006677 10.1039/d2sc05639a PMC10055764

[13] Wilkinson BL , Day S , Malins LR , et al. Self‐adjuvanting multicomponent cancer vaccine candidates combining per‐glycosylated MUC1 glycopeptides and the Toll‐like receptor 2 agonist Pam_3_CysSer. Angew Chem Int Ed Engl. 2011;50(7):1635‐1639. 21308921 10.1002/anie.201006115

[14] Gabba A , Attariya R , Behren S , et al. MUC1 glycopeptide vaccine modified with a GalNAc glycocluster targets the macrophage galactose C‐type lectin on dendritic cells to elicit an improved humoral response. J Am Chem Soc. 2023;145(24):13027‐13037. 37279388 10.1021/jacs.2c12843 PMC10288512

[15] Palitzsch B , Hartmann S , Stergiou N , et al. A fully synthetic four‐component antitumor vaccine consisting of a mucin glycopeptide antigen combined with three different T‐helper‐cell epitopes. Angew Chem Int Ed Engl. 2014;53(51):14245‐14249. 25318465 10.1002/anie.201406843

[16] Kaiser A , Gaidzik N , Becker T , et al. Fully synthetic vaccines consisting of tumor‐associated MUC1 glycopeptides and a lipopeptide ligand of the Toll‐like receptor 2. Angew Chem Int Ed Engl. 2010;49(21):3688‐3692. 20449837 10.1002/anie.201000462

[17] Apostolopoulos V , Osinski C , Mckenzie I . MUC1 cross‐reactive Galα(l,3)Gal antibodies in humans switch immune responses from cellular to humoral. Nat Med. 1998;4:315‐320. 9500605 10.1038/nm0398-315

[18] Kaiser A , Gaidzik N , Westerlind U , et al. A synthetic vaccine consisting of a tumor‐associated sialyl‐T(N)‐MUC1 tandem‐repeat glycopeptide and tetanus toxoid: induction of a strong and highly selective immune response. Angew Chem Int Ed Engl. 2009;48(41):7551‐7555. 19685547 10.1002/anie.200902564

[19] Westerlind U , Hobel A , Gaidzik N , et al. Synthetic vaccines consisting of tumor‐associated MUC1 glycopeptide antigens and a T‐cell epitope for the induction of a highly specific humoral immune response. Angew Chem Int Ed Engl. 2008;47(39):7551‐7556. 18704911 10.1002/anie.200802102

[20] Palitzsch B , Gaidzik N , Stergiou N , et al. A synthetic glycopeptide vaccine for the induction of a monoclonal antibody that differentiates between normal and tumor mammary cells and enables the diagnosis of human pancreatic cancer. Angew Chem Int Ed Engl. 2016;55(8):2894‐2898. 26800384 10.1002/anie.201509935

[21] Martinez‐Saez N , Peregrina JM , Corzana F . Principles of mucin structure: implications for the rational design of cancer vaccines derived from MUC1‐glycopeptides. Chem Soc Rev. 2017;46(23):7154‐7175. 29022615 10.1039/c6cs00858e

[22] Coelho H , Matsushita T , Artigas G , et al. The quest for anticancer vaccines: deciphering the fine‐epitope specificity of cancer‐related monoclonal antibodies by combining microarray screening and saturation transfer difference NMR. J Am Chem Soc. 2015;137(39):12438‐12441. 26366611 10.1021/jacs.5b06787

[23] Cai H , Sun ZY , Chen MS , et al. Synthetic multivalent glycopeptide‐lipopeptide antitumor vaccines: impact of the cluster effect on the killing of tumor cells. Angew Chem Int Ed Engl. 2014;53(6):1699‐1703. 24449389 10.1002/anie.201308875

[24] Martinez‐Saez N , Supekar NT , Wolfert MA , et al. Mucin architecture behind the immune response: design, evaluation and conformational analysis of an antitumor vaccine derived from an unnatural MUC1 fragment. Chem Sci. 2016;7(3):2294‐2301. 29910919 10.1039/c5sc04039f PMC5977504

[25] Braun P , Davies GM , Price MR , et al. Effects of glycosylation on fragments of tumour associated human epithelial mucin MUC1. Bioorg Med Chem. 1998;6(9):1531‐1545. 9801825 10.1016/s0968-0896(98)00092-3

[26] Fasting C , Schalley CA , Weber M , et al. Multivalency as a chemical organization and action principle. Angew Chem Int Ed Engl. 2012;51(42):10472‐10498. 22952048 10.1002/anie.201201114

[27] Ryan SO , Turner MS , Gariepy J , et al. Tumor antigen epitopes interpreted by the immune system as self or abnormal‐self differentially affect cancer vaccine responses. Cancer Res. 2010;70(14):5788‐5796. 20587526 10.1158/0008-5472.CAN-09-4519 PMC2905500

[28] Cai H , Zhang R , Orwenyo J , et al. Multivalent antigen presentation enhances the immunogenicity of a synthetic three‐component HIV‐1 V3 glycopeptide vaccine. ACS Cent Sci. 2018;4(5):582‐589. 29806004 10.1021/acscentsci.8b00060 PMC5968512

[29] Tirand L , Frochot C , Vanderesse R , et al. A peptide competing with VEGF165 binding on neuropilin‐1 mediates targeting of a chlorin‐type photosensitizer and potentiates its photodynamic activity in human endothelial cells. J Control Release. 2006;111(1‐2):153‐164. 16423422 10.1016/j.jconrel.2005.11.017

[30] Lei H , Shen Y , Song L , et al. Hapten synthesis and antibody production for the development of a melamine immunoassay. Anal Chim Acta. 2010;665(1):84‐90. 20381695 10.1016/j.aca.2010.03.007

[31] Paolino D , Licciardi M , Celia C , et al. Bisphosphonate‐polyaspartamide conjugates as bone targeted drug delivery systems. J Mater Chem B. 2015;3(2):250‐259. 32261945 10.1039/c4tb00955j

[32] Cai H , Huang ZH , Shi L , et al. Synthesis of Tn/T antigen MUC1 glycopeptide BSA conjugates and their evaluation as vaccines. Eur J Org Chem. 2011;2011(20‐21):3685‐3689.

[33] Zhou SH , Li YT , Zhang RY , et al. Alum adjuvant and built‐in TLR7 agonist synergistically enhance anti‐MUC1 immune responses for cancer vaccine. Front Immunol. 2022;13:857779. 35371101 10.3389/fimmu.2022.857779 PMC8965739

[34] Yu J , Song Y , Tian W . How to select IgG subclasses in developing anti‐tumor therapeutic antibodies. J Hematol Oncol. 2020;13(1):45. 32370812 10.1186/s13045-020-00876-4 PMC7201658

[35] Cabral MP , Garcia P , Beceiro A , et al. Design of live attenuated bacterial vaccines based on D‐glutamate auxotrophy. Nat Commun. 2017;8:15480. 28548079 10.1038/ncomms15480 PMC5458566

[36] Collins AM . IgG subclass co‐expression brings harmony to the quartet model of murine IgG function. Immunol Cell Biol. 2016;94(10):949‐954. 27502143 10.1038/icb.2016.65

[37] Du JJ , Zou SY , Chen XZ , et al. Liposomal antitumor vaccines targeting mucin 1 elicit a lipid‐dependent immunodominant response. Chem Asian J. 2019;14(12):2116‐2121. 31042017 10.1002/asia.201900448

[38] Yi M , Niu M , Xu L , et al. Regulation of PD‐L1 expression in the tumor microenvironment. J Hematol Oncol. 2021;14(1):10. 33413496 10.1186/s13045-020-01027-5 PMC7792099

[39] Pan J , Zeng W , Jia J , et al. A novel therapeutic tumor vaccine targeting MUC1 in combination with PD‐L1 elicits specific anti‐tumor immunity in mice. Vaccines (Basel). 2022;10(7):1092. 35891256 10.3390/vaccines10071092 PMC9325010

[40] Dai J , Hu JJ , Dong X , et al. Deep downregulation of PD‐L1 by caged peptide‐conjugated AIEgen/miR‐140 nanoparticles for enhanced immunotherapy. Angew Chem Int Ed Engl. 2022;61(18):e202117798. 35224832 10.1002/anie.202117798

[41] Bouillez A , Adeegbe D , Jin C , et al. MUC1‐C promotes the suppressive immune microenvironment in non‐small cell lung cancer. Oncoimmunology. 2017;6(9):e1338998. 28932637 10.1080/2162402X.2017.1338998 PMC5599083

[42] Nuhn L , Hartmann S , Palitzsch B , et al. Water‐soluble polymers coupled with glycopeptide antigens and T‐cell epitopes as potential antitumor vaccines. Angew Chem Int Ed Engl. 2013;52(40):10652‐10656. 24038824 10.1002/anie.201304212

[43] Cai H , Chen MS , Sun ZY , et al. Self‐adjuvanting synthetic antitumor vaccines from MUC1 glycopeptides conjugated to T‐cell epitopes from tetanus toxoid. Angew Chem Int Ed Engl. 2013;52(23):6106‐6110. 23616304 10.1002/anie.201300390

[44] Pett C , Cai H , Liu J , et al. Microarray analysis of antibodies induced with synthetic antitumor vaccines: specificity against diverse mucin core structures. Chem. 2017;23(16):3875‐3884. 10.1002/chem.201603921 27957769

[45] Yan S , Rolfe BE , Zhang B , et al. Polarized immune responses modulated by layered double hydroxides nanoparticle conjugated with CpG. Biomaterials. 2014;35(35):9508‐9516. 25145853 10.1016/j.biomaterials.2014.07.055

[46] Liu GH , Chen T , Zhang X , et al. Small molecule inhibitors targeting the cancers. MedComm. 2022;3(4):e181. 36254250 10.1002/mco2.181 PMC9560750

[47] Hou Y , Yan T , Cao H , et al. Chimeric hepatitis B virus core particles displaying Neisserial surface protein A confer protection against virulent Neisseria meningitidis serogroup B in BALB/c mice. Int J Nanomed. 2019;14:6601‐6613. 10.2147/IJN.S206210 PMC6702424 31496701

