# Supplementary material for: Synthetic self‐adjuvanted multivalent Mucin 1 (MUC1) glycopeptide vaccines with improved in vivo antitumor efficacy
Source: MedComm (2020). 2024 Feb 9;5(2):e484. doi: 10.1002/mco2.484 (PMC10857776; doi:10.1002/mco2.484)
Supplement: Supplementary file 1 — Supporting Information [file MCO2-5-e484-s001.docx]

Synthetic Self-Adjuvanted Multivalent Mucin 1 (MUC1) Glycopeptide Vaccines with Improved *in* *vivo* Antitumor Efficacy

Yang Zhou^1,#^, Xinru Li^1,#^, Yajing Guo^1^, Ye Wu^1^, Lixin Yin^1^, Luyun Tu^1^, Sheng Hong^1^, Hui Cai^1,*^, Feiqing Ding^1,*^

^1^School of Pharmaceutical Sciences (Shenzhen), Shenzhen Campus of Sun Yat-Sen University, 66 Gongchang Lu, Guangming District, Shenzhen 518107, China

**∗Correspondence**

Hui Cai, School of Pharmaceutical Sciences (Shenzhen), Shenzhen Campus of Sun Yat-Sen University, 66 Gongchang Road, Guangming District, Shenzhen 518107, China. Email: [caihui5@mail.sysu.edu.cn](mailto:caihui5@mail.sysu.edu.cn)

Feiqing Ding, School of Pharmaceutical Sciences (Shenzhen), Shenzhen Campus of Sun Yat-Sen University, 66 Gongchang Road, Guangming District, Shenzhen 518107, China. Email: [dingfq3@mail.sysu.edu.cn](mailto:caihui5@mail.sysu.edu.cn)

^#^Yang Zhou and Xinru Li contributed equally to this work.


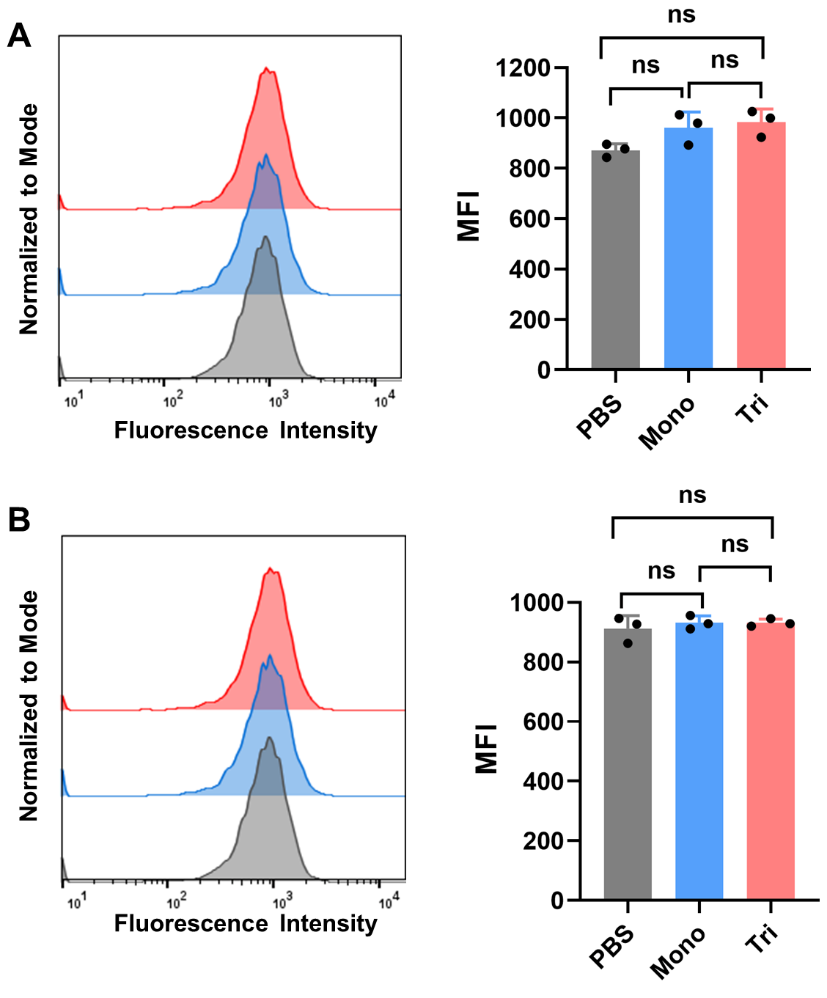


**Figure S1.** Antibodies from the vaccinated mice showed no binding to HEK293T cells. Flow cytometry analysis of the binding ability of the antisera (1:100 dilution) to HEK293T cells from WT (**A**) and Tg (**B**) mice. PBS: incubation of the cells with sera from the mice that were not immunized.


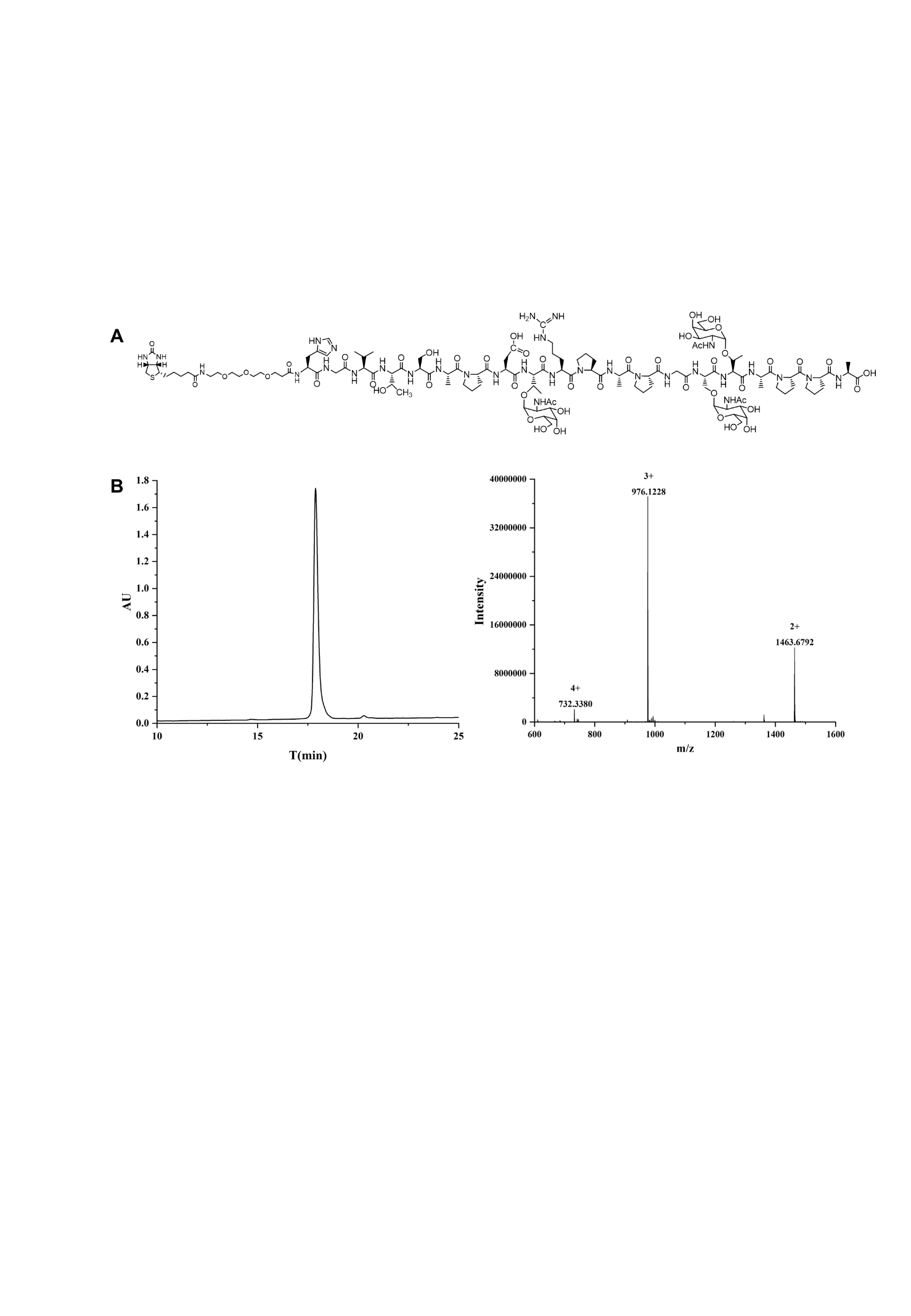


**Figure S2.** Structure and characterization of the biotinylated MUC1 glycopeptide used in ELISA. Structure of the biotinylated MUC1 glycopeptide **(A)**, and the analytical HPLC profile (left panel) and HRMS spectrum (right panel) of it **(B)**.
